# Ratio of carbon and nitrogen in fertilizer treatment drives distinct rhizosphere microbial community composition and co-occurrence networks

**DOI:** 10.3389/fmicb.2022.968551

**Published:** 2022-09-08

**Authors:** Ruifen Zhu, Chang Liu, Yuan Dong Xu, Wei He, Jielin Liu, Jishan Chen, Yajun An, Shangli Shi

**Affiliations:** ^1^Pratacultural College, Gansu Agricultural University, Lanzhou, China; ^2^Pratacultural Institute, Chongqing Academy of Animal Sciences, Rongchang, China; ^3^Pratacultural Institute Science, Heilongjiang Academy of Agricultural Sciences, Harbin, China; ^4^Gansu Yasheng Agricultural Research Institute Co., Ltd., Lanzhou, China

**Keywords:** ratio of carbon and nitrogen, rhizosphere, microbial community composition, co-occurrence networks, alfalfa

## Abstract

Fertilization is the main strategy to accelerate vegetation restoration and improve the rhizosphere microbial community in the northeast China. However, the responses of rhizosphere microbial community structure, specific microbial community and symbiotic pattern to manure fertilization in grassland (alfalfa only) are not well clear. In this study, the variation of bacterial community structures in R_Manure (extracted liquid of fermented cow manure), E_Manure (extracted residue of fermented cow manure), F_Manure (full fermented cow manure), and Control (without fermented cow manure) collected from the rhizosphere microbial community of alfalfa were analyzed by the application of an Illumina HiSeq high-throughput sequencing technique. A total of 62,862 microbial operational taxonomic units (OTUs) were detected and derived from 21 phyla of known bacteria. The dominant bacteria in the rhizosphere include Proteobacteria (70.20%), Acidobacteria (1.24%), Actinobacteria (2.11%), Bacteroidetes (6.15%), Firmicutes (4.21%), and Chlorofexi (2.13%) accounting for 86% of the dominant phyla in all treatments. At the genus level, the dominant genus include *NB1-j, Lysobacter, Alphaproteobacteria, Subgroup_6, Actinomarinales, Saccharimonadales, Aneurinibacillus, MO-CFX2, SBR1031, Caldilineaceae, and so on* with the average relative abundance (RA) of 1.76%, 1.52%, 1.30%, 1.24%, 1.61%, 2.39%, 1.36%, 1.42%, 1.27%, and 1.03%, respectively. Bacterial diversities and community structures were significantly differentiated by different treatments of fertilization. The results of community structure composition showed that R_Manure treatment significantly increased the population abundance of Firmicutes, Chlorofexi, and Patescibacteria by 34.32%, 6.85%, and 2.70%, and decreased the population abundance of Proteobacteria and Actinobacteria by 16.83% and 1.04%, respectively. In addition, it showed that all treatments significantly resulted in an increase or decrease at the genus level. R_Manure had the higher richness and diversity of the bacterial community, with the greatest topology attributes of the co-occurrence networks. Through the analysis of the molecular ecological network (MENA), the co-occurrence networks had a shorter average path distance and diameter in R_Manure than in others, implying more stability to environmental changes. Redundancy analysis (RDA) showed that the ratio of carbon and nitrogen (C/N) was the main factor affecting rhizosphere microbial community composition while driving distinct rhizosphere bacterial community and its co-occurrence networks. The R_Manure associated with more C/N had relatively complex microbial co-occurrence network with a large number of nodes and edges, while the microbial network of others associated with less C/N had fewer taxa with loose mutual interactions. These results suggested that organic fertilizer with high C/N can regulate the rhizosphere microorganism, while high C/N can determine bacterial community structures, specific bacterial taxa, and their relationships with the nodule size of alfalfa. These significant changes can be used to evaluate soil fertility and fertilizer management in the artificial grassland system, while the potential biological indicators of the rhizosphere microbial community will play an important role in future eco-agriculture.

## Introduction

Soil microorganisms play an important role in terrestrial nutrient cycling processes such as C and N cycles. Their decomposition and participation play a part in the nutrient cycle of terrestrial plants, which are very important to plant growth and health (Kennedy, 1999; Marschner et al., 2004; Osler and Sommerkorn, 2007), especially some plant's rhizospheric and endogenous microorganisms can improve plants tolerance to abiotic and biotic stresses (Mendes et al., 2013). Therefore, agricultural productivity may be determined by the rhizosphere microorganisms through altering bacterial activities, compositions, or interactions (Heijden et al., 2008). To a great extent, the changes in soil bacterial communities may affect agricultural ecosystem processes. However, the responses of the structural characteristics and molecular ecological network of the rhizosphere microbial communities to specific environmental factors induced by nutrient amendments are not clear.

Increasing attention has focused on the significance of rhizosphere microbial communities in many research reports (Heijden et al., 2008; Song et al., 2018; Tripathi et al., 2018), while the loss of soil microbial diversity is considered to be the main factor threatening the ecosystem balance. In addition, the composition of rhizosphere microbial communities in different ecosystem biological communities is quite different (Song et al., 2018; Tripathi et al., 2018). For example, one study reported that the distribution of microbial community depends on latitude gradient (Zhang et al., 2016), while other studies have shown that the distribution of microbial community also depends on altitude gradient (Yang et al., 2014; Zhang et al., 2015), soil pH (Shen et al., 2013; Tripathi et al., 2018), soil nutrient (Zhang et al., 2017), and climate temperature (Davidson et al., 2006). In fact, more man-made factors currently disrupt the functioning of terrestrial ecosystems and continue to destroy the composition of soil microorganisms, such as inappropriate fertilization (Walther et al., 2002; Zhang et al., 2018). In contrast, little is known about how rhizosphere microbial community composition and co-occurrence networks respond to the fertilized grassland ecosystems, especially in the rhizosphere of Alfalfa.

Drenovsky et al. (2010) show that fertilization is closely related to different microbial communities at the regional level. Recently detailed descriptions of soil microbial communities have shown that fertilization affects different patterns of soil microbial communities in natural forests, including vegetation gradients and different soil variables (Ma et al., 2017). Similarly, different microbial diversity patterns have been observed in different ecosystems of drylands in northern China, such as alpine grassland, desert grassland, and typical grassland (Wang et al., 2017). In the meantime, more and more surveys show that the fertilization strategies got a lot of attention, while crop yields and soil quality including soil microbial biomass, activities, and community structure are intensively studied (Fierer et al., 2007). However, the effects of short-term C and N fertilization on the rhizosphere microbial community remain largely unknown.

The imbalance of soil microbial diversity and function caused by fertilization has aggravated the degradation of the soil ecosystem (Sala et al., 2000; Singh et al., 2011; Verhulst et al., 2011). Previous studies have shown that fertilization, especially chemical fertilizer, has a significant impact on soil bacterial community structure and composition (Meriles et al., 2009; Aziz et al., 2013; Navarro-Noya et al., 2013). Many studies have also shown the effects of soil parameters (such as pH value, electrical conductivity, carbon and nitrogen content, salinity, and texture) on microbial community composition (Chaudhry et al., 2012; Bartram et al., 2014; Hartmann et al., 2015; Min et al., 2016). This relationship is very important even in unique environments in Northeast China (Lauber et al., 2008).

Therefore, soil bacterial taxa with different relative abundance (RA) patterns are considered to be potential biological indicators reflecting soil environmental conditions (Tripathi et al., 2014). A recent study shows that soil microbial community structure shifted after fertilization, while a significant difference in rhizosphere community structure was found between mineral fertilizer soils (P, N, and NP) and manure application soils (M, NM, PM, and NPM) (Wang et al., 2016). They also reported that some dominant microbial communities were markedly correlated with particular soil parameters. These results support the use of particular bacterial taxa and their RA as biological indicators for predicting various soil physical and chemical properties (e.g., soil pH value and soil nutrient concentration).

To reveal the complex interaction among microbial communities, a co-occurrence network has been widely used in the analysis of various microbial communities (Bartram et al., 2014; Hartmann et al., 2015). In this network, keystone taxa that interact frequently with many other species are expected to play an important role in microbial molecular ecology (Lauber et al., 2008; Tripathi et al., 2014; Min et al., 2016). Different co-occurrence networks have been shown in different fertilization practices (organic and traditional agriculture) (Lauber et al., 2008) and habitats (root zone and rhizosphere) (Lauber et al., 2008; Wang et al., 2016). However, the changes in the symbiotic networks of bacterial communities in the rhizosphere of alfalfa fertilized with manure have not been explored, while the links between manure application correlated to C and N contents or C:N ratio and rhizosphere microbial communities are not well-understood.

This study focuses on the impact of fermented cow manure fertilization on the rhizosphere microbial community of alfalfa and the responses of alfalfa rhizosphere microbial communities to the separation liquid of fermented cow dung, the separation residue of fermented cow dung, and the fermented cow dung which is a typical microenvironment facing the different ratio of carbon and nitrogen (C/N) inherent cow dung fertility. So far, there are still deficiencies in microbial diversity and co-occurrence patterns of alfalfa rhizosphere microbial communities under the conditions of organic fertilizer. Therefore, the contents of our research are as follows: (1) to investigate the characteristics of bacterial communities of alfalfa rhizosphere in the condition of four different treatments with fermented cow manure; (2) to compare the microbial diversity and co-occurrence patterns of the bacterial community; (3) to identify key driver factors of fertilization with cow manure affecting microbial diversity and co-occurrence networks.

## Materials and methods

### The experiment site description

Our experiment was in a normal greenhouse management carried out at the Frigid Forage Research Station. The station has an altitude of 160 m, longitude of 125°28′24″E, and latitude of 46°32′17″N in northeast China. The experiment site occupies a sub-humid climate and a dark loam (mostly Chernozem, FAO Taxonomy) (Chen et al., 2013). Because of an irreversible trend of degradation in this region, degraded meadows characterized by hybrid grass needed reseeding (alfalfa) methods for vegetation restoration. In fact, it was significantly better using cow manure in RG (reseeding grassland) than without cow manure in NG (natural grassland). To reveal the effect of cow manure on alfalfa (*Medicago sativa*) forage, we designed a fully controllable single-factor experiment to investigate the effects of the components from cow manure under the same condition (normal greenhouse), while providing an ideal platform to examine the carbon effects on rhizosphere microbial community and co-occurrence patterns. More descriptions were reported in the published article by Zhu et al. (2020).

### Experimental design and treatment description

The pot experiment in the greenhouse was initiated in May 2019. We selected alfalfa (*Medicago sativa* L. Gannong No. 5 provided by the key Laboratory of Pratacultural Ecosystem, Ministry of Education, Gansu Agricultural University, with 97% clarity and 95% germination rate) as the dominant species in our experimental pots, and designed the randomized block trials with four treatments of fertilization and 12 replicates for each treatment. To survey rhizosphere bacterial communities and co-occurrence patterns in the condition of fertilization with fermented cow manure, we selected the fermented cow manure purchased from the specialized company of the Xiaoliugu farm and separated it as follows: First, soak a certain amount of fermented cow manure purchased with sterile water in a ratio of 1:6 (mixed 129 g cow manure and 750 ml sterile water) for 24 h and keep stirring every hour to keep the mixture cool and smooth. After that, solid-liquid separation was done. The solid part (self-absorption water of 50 ml) is manure residue (R_Manure) and the liquid part (700 ml) is manure extract (E_Manure).

Before the pot experiment, the sands were used as the main matrix, which was purchased from the local market, which needs to be cleaned and the pH adjusted to neutral and dry. Then, equal portions (3.5 kg with a volume of 0.0028 m^3^) of sands were put into the plastic flowerpot with a diameter of 18 cm and a volume of 0.0028 m^3^ (Supplementary Figure S1), respectively. According to the two parts separated from fermented cow manure as mentioned above, the treatment of R_Manure stands for adding the solid part and the treatment of E_Manure stands for adding the liquid part (700 ml). In parallel, the treatment of F_Manure stands for adding the unseparated fermented cow manure (129 g/pot) and Control stands for without adding fermented cow manure. All treatments including R_Manure, E_Manure, F_Manure (unseparated fermented cow manure), and Control (without fermented cow manure) were arranged in the pot trials.

For keeping the same amount of water and basic nutrients before sowing, all treatments were unified under the water condition of 700 ml. The sterile water of 650 ml was used in the treatment of R_Manure (self-absorption water of 50 ml) while the sterile water of 700 ml was used in the treatment of F_Manure, and 700 ml Hoagland nutrient solution was used in the treatment of Control. The substrate of our pot experiment is sandy rather than soil. See Supplementary Figure S1 for a detailed description.

We selected the full and healthy seeds of alfalfa for sterilization for 3 min with iodophor solution (effective iodine content 0.45–0.55%) in a biosafety cabinet, then washed with sterile water for 5–6 times and dried. After that, we put the seeds into the flowerpot and sow 10 seeds per pot at a depth of 3 cm. When the seeds emerge completely, the alfalfa seedlings are thinned from 10 to 8 plants per pot according to 330 plants per m^2^ in the alfalfa production field. Throughout the trial period, uniform indoor culture conditions followed were described as follows: the illumination (7,000–8,000 lux), the illumination time (12 h/day), the temperature (21–25°C) with illumination, the temperature (16–20°C) without illumination, and the relative humidity (50–70%).

During the peak period of vegetation growth (120 day), rhizosphere samples were collected from the pots treated with separated residue of cow manure (R_Manure), separated liquid of cow manure (E_Manure), cow manure (F_Manure), and without cow manure (Control). In each treatment, we gathered the surface soil of alfalfa root samples (soil attached to the root about 1 mm thick is defined as rhizosphere soil) from each pot (80 g from each pot), while stored at −80°C immediately for later DNA analysis. Additionally, we gathered the matrix (sands) and air-dried in the room for 15 days and sieved them to analyze the properties.

### The physico-chemical properties

The physical properties of the matrix (mixed sands) were examined following the procedures described in Bao (2000). The other subsample was sieved (2-mm mesh) to remove roots and stones, and thereafter dried for chemical analyses. Total N in the matrix in each plot was determined with FOSS Kjeltec 2300 Analyzer Unit (FOSS, Hillerød, Sweden). For total phosphorus (P), we followed the sodium hydroxide melting molybdenum antimony colorimetric method (Yang et al., 2018). Available P was also determined using a Flow-Solution analyzer (Flowsys, Ecotech, Germany).

### High-throughput sequencing

High-throughput sequencing was performed using a method described in Zhou et al. (1996). In brief, DNA was extracted from 0.5-g soil samples (−80°C) by using the MP FastDNA spin kit for soil (MP Biomedicals, Solon, OH, USA) according to the manufacturer's instructions. We used a Nano DropTM 2000 spectrophotometer (Thermo Fisher Scientific, Wilmington, MA, United States) for DNA concentration and purity at A260/A280 and A260/A230. We amplified a region of the 16S rRNA gene for archaea by 515F (5′-GTGCCAGCMGCCGCG GTAA-3′)/806R(5′-GGACTACHVGGGTWTCTAAT-3′) according to the conservative region design, then added sequencing connectors at the end of the primers, conducted PCR amplification, and purified, quantified, and homogenized the products to form a sequencing library. The established library was first tested for library quality, and Illumina HiSeq was used to sequence the qualified library. The original image data files obtained by high-throughput sequencing (Illumina HiSeq and other sequencing platforms) were analyzed and converted into Sequenced Reads by Base Calling. All samples are amplified in triplicate, and no template controls are included in all the steps of the process. Triplicate PCR amplicons were pooled and then subjected to electrophoresis detection in 2% (W/V) agarose gels. PCR products with bright bands were mixed at an equal density ratio and purified with GeneJET Gel Extraction Kit (Thermo Scientific). The purified PCR amplification products were sequenced on the Illumina MiSeq (300-bp paired-end reads) platform (Illumina Inc., San Diego, USA) at Novogene Bioinformation Technology Co., Ltd., San Diego, USA. Obtained sequences were quality filtered, and any chimeric sequences were removed using the USEARCH tool based on the UCHIME algorithm. Sequences are grouped according to their taxonomy and assigned to operational taxonomies (OTUs) at varying levels of 3% by using the UPARSE pipeline. Those OTUs with less than two sequences were removed, and their representative sequences were classified within the SILVA database release 128 for bacteria and archaea.

### Statistical analysis

Based on the high-throughput sequencing of bacteria, Illumina HiSeq was used (Deng et al., 2012). The website of the University of Oklahoma (http://ieg2.ou.edu/MENA) was used to construct the molecular ecological network of the microorganisms with different fertilization treatments and to calculate its topological parameters. According to the random matrix theory (Zhou et al., 2010, 2011), the adjacency matrix was derived from the similarity matrix by applying the appropriate threshold value, and the adjacency matrix was used to encode the connection strength between each pair of nodes. The topology parameters are used for the description of the network information. Gephi 0.9.1 software was used to draw the network diagram and compare the structure of the microbial network in the four fertilization treatments.

All data analyses including analysis of variance for soil properties and PERMANOVA analysis for microbial diversity were done with the R software (Ogle, 2017). Microbial diversity was also shown by Vegan packages in R software. The characteristics of microbial communities were sequenced by principal components analysis (PCA). The relationship between community distribution patterns and environmental factors was estimated by the redundancy analysis (RDA) (R Core Team., 2013). Further statistical analysis was carried out using other packages in R software, while the molecular ecological network was visualized using the software of Gephi 0.9.1 (Bastian et al., 2009).

## Results

### Bacterial community variation among the treatments of fertilization

**Figure 1:**
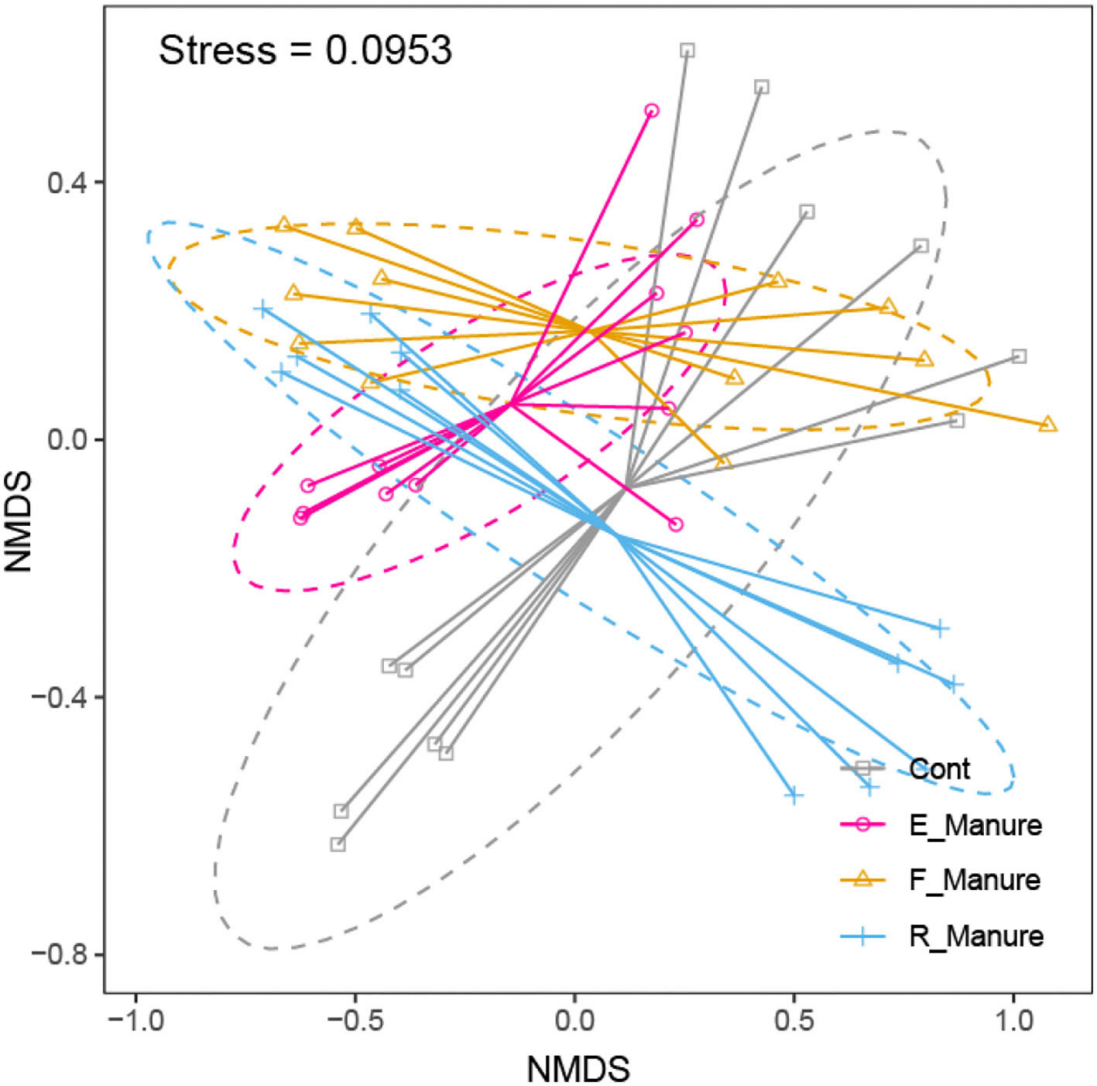
Non-metric multidimensional scaling (NMDS) ordination of bacterial communities. PERMANOVA shows that fertilization effects are the strongest, contributing the largest components of variation to the overall model (*p* = 0.001 < 0.05).

**Figure 2:**
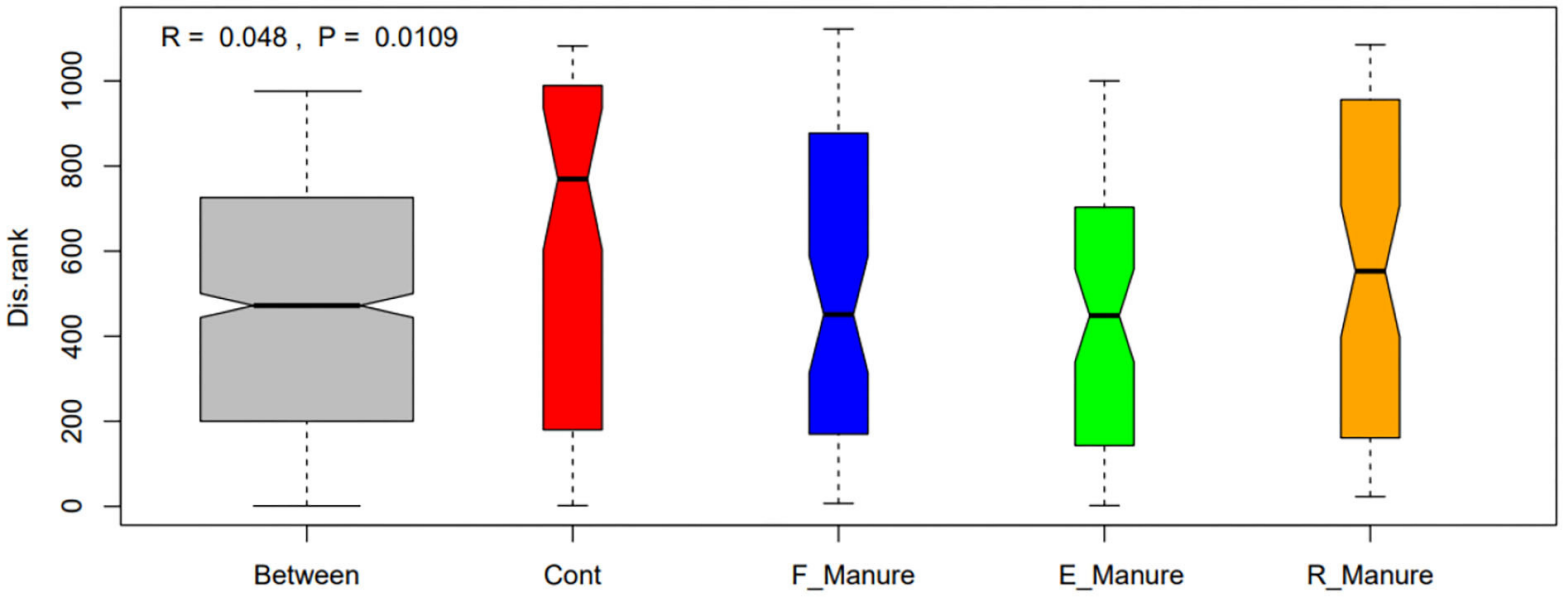
The beta dispersion of bacterial communities in the four treatments of fertilization by box plot illustration. Significant differences between treatments of fertilization were tested by ANOSIM and are indicated by the value range of *R*-value (anosim statistical R) and *P*-value (significance). *R*-value is (−1,1). *R* > 0 indicating that the difference between groups is greater than that within groups, that is, the difference between groups is significant; *R* <0 indicating that the difference within the group is greater than that between the groups. The greater the absolute value of *R*, the greater the relative difference. The lower the *p*-value, the more significant the difference in test result is. Generally, 0.05 is the significance level limit. Boxes represent the interquartile range (IQR), and whiskers indicate the furthest point within 1.5 × IQR above or below the IQR. Values beyond this range are plotted as individual points. The central line indicates the median.

Our study obtained a total of 3,616,347 high-quality sequences by Illumina HiSeq of 16S rRNA gene, and operational taxonomic units (OTUs) of 62,862 were identified based on 97% sequence consistency. Based on a non-metric multidimensional scale (NMDS), rhizosphere bacterial communities of E_Manure, R_Manure, and F_Manure were significantly different from those of the Control treatment (Figure 1). PERMANOVA showed that fertilization effects are the strongest, contributing the largest components of variation to the overall model (the overall model means the whole level and not two-by-two groups, *R*^2^ = 0.6737, *p* = 0.001 <0.05). Significant differences between different treatments were certified by pair similarity comparison analysis (ANOSIM) and permutation multiple variance analysis (PERMANOVA) (Supplementary Table S1). Although the bacterial communities of the F_Manure and E_Manure were close together in the sequenced blocks (Figure 1), pair tests were significant for all treatments (Supplementary Table S1). The distribution of rhizosphere bacterial communities in different components separated from fermented cow dung was studied by determining the distance between centroids. The beta dispersion of bacterial communities among different treatments was the lowest in E_Manure and the highest in R_Manure (Figure 2). To compare alpha-diversity among different treatments, OTU data were analyzed with seven indicators (Table 1). Chao1 and ACE of E_Manure were significantly higher than that of the Control. Similarly, Chao1 and ACE of F_Manure and R_Manure were significantly higher than those of the Control. The results showed that compared with the Control, the richness of bacterial community in other treatments was significantly increased.

**Table 1:** Alpha diversity for bacterial communities in the four treatments of fertilization.

**Treatment**	**Richness**	**Shannon**	**Pielou**	**Simpson**	**Equitability**	**Chao1**	**Ace**
Control	994.33^c^	5.41	0.78	87.75	0.09	1077.35^c^	1055.52^c^
F_Manure	1111.67^b^	5.34	0.76	78.26	0.07	1227.97^b^	1211.37^b^
E_Manure	1263.67^a^	5.58	0.78	96.06	0.08	1335.79^a^	1313.34^a^
R_Manure	1164.33^b^	5.51	0.78	87.28	0.07	1228.10^b^	1221.66^b^

^a−*c*^The letters in each column indicate significant differences (P < 0.05).

### Variation in the chemical properties among the treatments of fertilization

**Figure 3:**
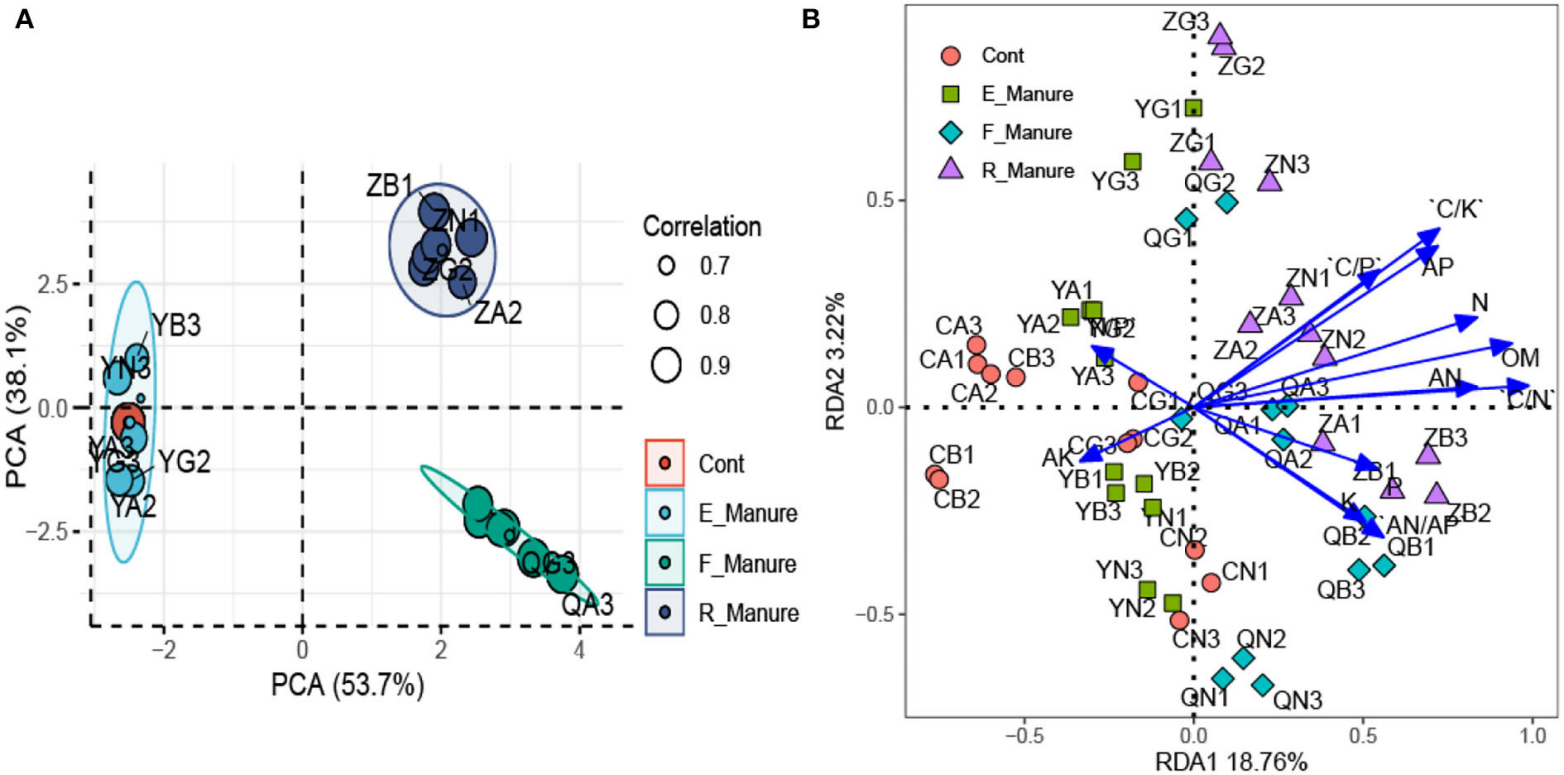
The chemical properties associated with the treatments of fertilization. Principal component analysis (PCA) of chemical properties using z-transformed variables **(A)**. Redundancy analysis (RDA) of bacterial communities constrained by the chemical properties **(B)**. The joint biplot indicates the correlation between the chemical factors and ordination scores of RDA axes. C/N, ratio of carbon and nitrogen; OM, organic matter.

Based on the PCA, the chemical properties shown by the ordination plot were distinctly separated between R_Manure and F_Manure along the first axis, which explains 53.7% of the total variation, and those in the rhizosphere of E_Manure and Control were explained in between 38.1% (Figure 3A). Of the matrix properties value measured, organic matter (OM), available phosphorus (AP), and available nitrogen (AN) were 3.57%, 57.14%, and 166.67% significantly higher in R_Manure than the matrix of other treatments, while the Control matrix had significantly lower values of nitrogen (N), phosphorus (P), and potassium (K) than those in other substrates (Table 2). Based on the RDA constrained by the ordination plot, the chemical properties of the matrix also showed that bacterial communities were separated by different treatments along the first axis (Figure 3B). The chemical properties measured explained 21.98% of the total variation in our study. For each soil property, the C/N with the highest conditional effects (0.02767, *p* < 0.001) and marginal effects (0.01321, *p* < 0.001) are the main factors shown by the biplots, while playing an important role in the dispersion of the bacterial communities along the first axis. The specific OTUs we identified are distinctly correlated with the chemical properties.

**Table 2:** Description of the chemical properties in the four different treatments.

	**N** **g/kg**	**P** **g/kg**	**K** **g/kg**	**AN** **mg/kg**	**AK** **mg/kg**	**AP** **mg/kg**	**OM** **g/kg**	**C/N**	**C/P**	**C/K**	**N/P**	**AN/AP**
Control	0.11^b^	0.02^c^	0.14^b^	0.02^b^	0.23	0.04^b^	0.77^b^	7.14^c^	43.58^b^	5.56^c^	6.10^a^	0.60^c^
F_Manure	2.79^a^	1.68^a^	2.14^a^	0.16^a^	0.24	0.06^b^	42.92^a^	15.78^b^	27.63^c^	20.45^b^	1.76^b^	2.87^a^
E_Manure	0.11^b^	0.02^c^	0.14^b^	0.02^b^	0.23	0.04^b^	0.77^b^	7.33^c^	44.20^b^	6.26^c^	6.06^a^	0.61^c^
R_Manure	2.44^a^	0.38^b^	0.34^b^	0.14^a^	0.18	0.14^a^	44.51^a^	18.60^a^	120.34^a^	133.23^a^	6.47^a^	1.02^b^

C/N, ratio of carbon and nitrogen; OM, organic matter.

^*a*−*c*^The letters in each column indicate significant differences (P < 0.05).

### Indicator taxa for specific treatments of fertilization

**Figure 4:**
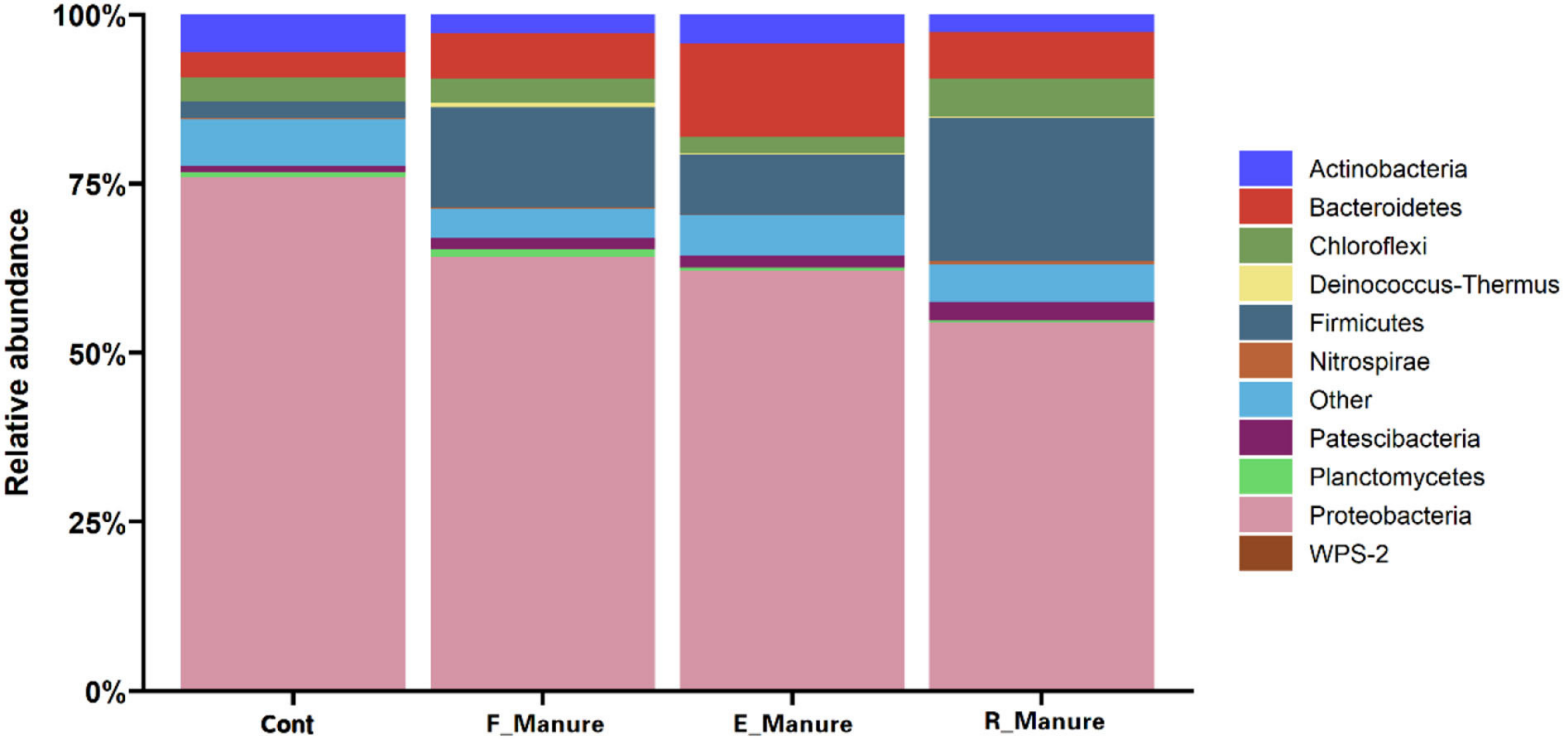
Taxonomic distribution of the bacterial communities in the four treatments of fertilization. The phyla with an abundance of <1% are indicated as “others.” For Proteobacteria, the classes are not indicated. The stacked column bar graph was generated using R software.

The 62,862 OTUs were obtained from all samples in our treatments, while 97.52% OTUs were assigned to phylum-level taxa. In this study, the 21 phyla identified had RA > 1% and accounted for 94.29% of the total abundance, with Proteobacteria (64.13%), Firmicutes (11.81%), Bacteroidetes (7.79%), Chloroflexi (3.81%), and Actinobacteria (3.79%) being the dominant phyla of the bacterial communities across the different treatments (Figure 4). Relative abundance of Proteobacteria (75.91%) was higher in the Control than in others, while *Firmicutes* (14.78% and 21.19%, respectively) in F_Manure and R_Manure were more abundant than in the other two treatments. The RA of Bacteroidetes (13.85%) in E_Manure was about three times higher than the Control, while the RA of Chlorofexi (2.34%) was lower than those of the other treated matrix.

**Figure 5:**
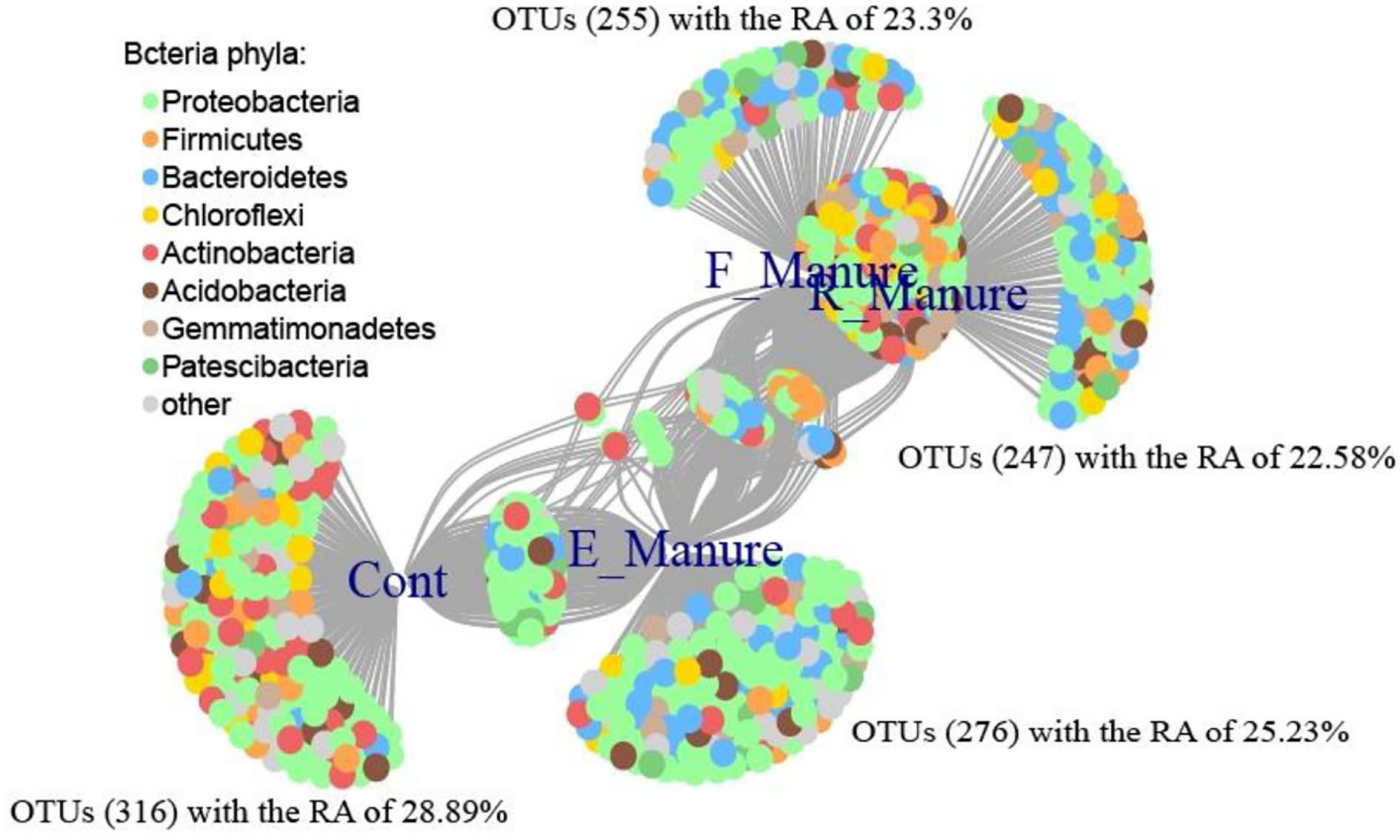
Bipartite network showing the associations between the four treatments of fertilization and 1,094 significantly associated OTUs (*P* < 0.01). Edges (node connection) show the association of individual OTUs with each treatment of fertilization. OTUs are colored by phylum. The network analysis was visualized using R.

Based on point biserial correlation, indicator species analysis was performed to identify individual OTUs sensitive to the different treatments. The 1,094 OTUs were significantly correlated with the different treatments or their combinations in the condition of point biserial correlation coefficient *R* > 0.6 and *P* < 0.001, which were clearly shown by a bipartite network (Figure 5). The sequence reads of these indicator OTUs accounted for 1.78% of the total number of sequences. The Control holds the most indicator OTUs (316), with an RA of 28.89%, followed by E_Manure (276 OTUs, with an RA of 25.23%), F_Manure (255 OTUs, with an RA of 23.3%), and R_Manure (247 OTU, with an RA of 22.58%), indicating that R_Manure provides a less distinctive niche than the other treatments which use indicator species analysis. The OTUs, consisting the indicator taxa in E_Manure, belong mainly to the phyla Proteobacteria and Bacteroidetes, and those of the indicator taxa in F_Manure belong mainly to the phylum Firmicutes and the class Alphaproteobacteria. The R_Manure had the indicator taxa belonging to Firmicutes and the class Alphaproteobacteria, in particular, Chloroflexi and the phylum Nitrospirae. The OTUs consist of the indicator taxa in Control also were belong mainly to the phylum Actinobacteria and the class Alphaproteobacteria, but only one specific indicator taxon which belonged to the genus *Deinococcus-Thermus*.

From the indicator taxa of each different treatment, the sensitive OTUs (csOTU) were detected including OTU4, OTU1104, OTU178, OTU81, OTU14, OTU629, OTU102, OTU126, OTU8420, and OTU143 (Supplementary Table S2). OTU4, OTU102, OTU1104, and OTU14 were related to Chloroflexi and clustered with uncultured bacterial clones detected in R_Manure. OTU126 is widely distributed in all fertilization treatments. OTU81 and OTU629 were connected with Gammaproteobacteria and clustered with uncultured bacterial clones shown in all treated matrix (mixed sands). OTU8420 and OTU143 belong to Alphaproteobacteria and was clustered with uncultured bacterial clones shown in all treated matrix (mixed sands). In conclusion, the majority of bacterial communities were not differentiated by the different treatments, while there were distinct taxa specific to the different fertilization treatments.

### Co-occurrence networks of bacterial communities among the treatments of fertilization

**Figure 6:**
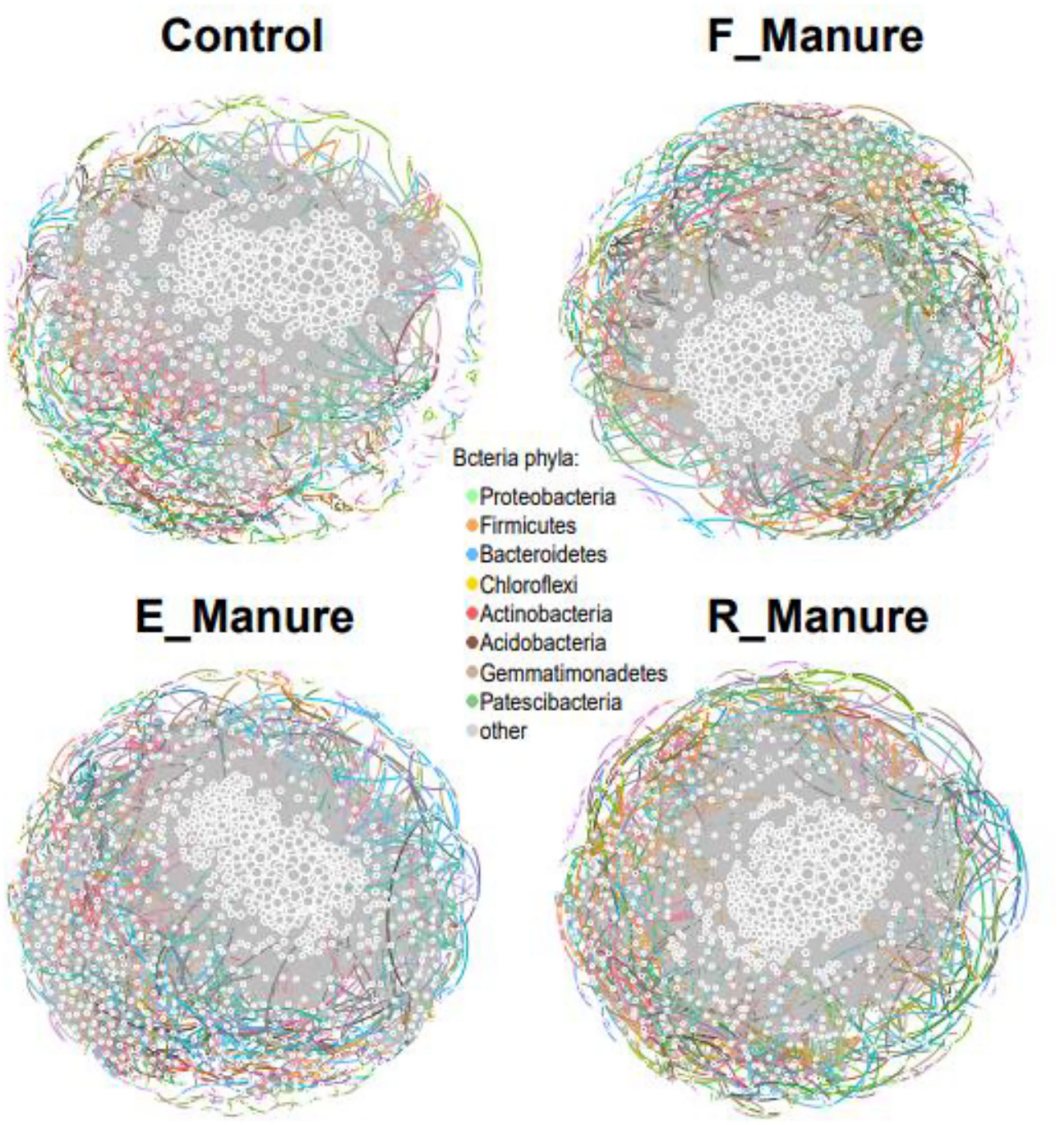
Co-occurrence networks of each treatment of fertilization. Circles indicate OTUs associated with the network. In particular, the size of circles and triangles is proportional to the number of degrees. OTUs are colored by phylum. The network analysis was visualized using Gephi 0.9.1. Topological properties of the networks of bacterial communities in four different fertilization treatments were shown and others in Supplementary Table S3.

Based on the random-metric theory (RMT), co-occurrence networks analysis using molecular ecological network analyses (MENA) was performed to explore the complex microbial community structures. In the analysis of the four treatments, common OTUs present in more than 60% of samples were used. The network connectivity with a high level of *R*^2^ of power-law showed scale-free properties (Supplementary Table S3). The number of OTUs related to the networks was the highest in R_Manure and the lowest in Control (Figure 6). The average network distance, referred to as the average path length (GD), was the highest in the network of Control. However, the connectivity between OTUs, referred to as average degree (avgK), was the highest in the network of R_Manure, followed by E_Manure, F_Manure, and Control. The results of network topology showed that relatively large numbers of bacterial taxa in the bacterial communities of R_Manure were related with the co-occurrence networks which tended to be closely connected to each other, and the inverse of relatively low numbers of OTUs tended not densely connected to each other.

Further analysis showed that OTUs in a microbial ecosystem have the most frequent interactions with other taxa and the highest value of betweenness centrality in the co-occurrence networks, which are named as potential keystone OTUs (Supplementary Table S4). The keystone OTUs detected in this study also varied with the different treatments: OTU4, OTU14, OTU102, and OTU1104 (phylum Chlorofexi) in R_Manure; OTU81 and OTU629 (class Gammaproteobacteria) in the Control; OTU8420 and OTU143 (class Alphaproteobacteria) in F_Manure; OTU404 (class *Bacilli*) in R_Manure and F_Manure; OTU126 (phylum Actinobacteria) in Control; and OTU178 (phylum Acidobacteria) in F_Manure. Consistently, none of the keystone OTUs identified have been highly abundant in all the treatments. In fact, most of the keystone OTUs in the networks showed relatively less connectivity with other OTUs (Supplementary Tables S3, S4, Figure 6), suggesting that keystone OTUs with high connectivity are independent of indicator taxa.

## Discussion

In this study, the fertilization treatments significantly affected rhizosphere bacterial diversities and community structures, shown in Figures 1, 2 and Table 1, which were suggested as the chemical properties of which R_Manure and F_Manure exhibited the most distinct characteristics in comparison with the other treatments; E_Manure also had variant characteristics from those of Control treatment (Table 2). The subset of bacterial taxa was specific to each treatment of fertilization, which was related to different phylum distributions in the bacterial communities (Figures 3, 4). The bacterial communities in different treatments of fertilization exhibited distinct co-occurrence networks (Figure 6, Supplementary Table S3).

### Treatments of fertilization affect bacterial community structures

From the NMDS analysis we observed (Figure 1), our findings are consistent with similar results reported by previous studies (Morlon et al., 2008; Drenovsky et al., 2010; Figuerola et al., 2012; Jiménez-Bueno et al., 2016), which support that bacterial distribution is significantly different in different treatments at grassland scales. In fact, different land use, such as alfalfa field, other crop fields, and so on, also affects soil chemical properties (Liesack et al., 2000). In this study, alfalfa fields are unique environments as a perennial grassland, which has homogeneous soil structure and properties, and supporting the high yield of aboveground alfalfa (the hay yield is about 9,750–10,500 kg/hectare). So the components separated from fermented cow manure, including R_Manure and E_Manure, influence matrix (mixed sands) compartments and microbial communities, resulting in different bacterial diversities, such as richness, Chao1, and Ace in the four treatments of fertilization (Table 1), including the most remarkable community structure from those of the other fertilization gradients (Figure 1). As we know, bacterial richness and diversity are highest in neutral soils and lower in acidic soils (Ma et al., 2017). This is in agreement that the unimodal diversity patterns in soils with different fertilization treatments were also observed. R_Manure and F_Manure will lead to an accumulation of chemical components in the matrix (sand), such as OM, AP, and the C/N, which inputs to enhance alfalfa productivity. This relates to the distinct chemical properties of matrix treated with fermented cow manure indicated by the joint biplot (Figure 3). These chemical factors we knew had been reported in terms of affecting microbial community structures (Reich et al., 2005; Handley et al., 2012; Sridevi et al., 2012; Kang et al., 2013; Singh et al., 2013; Ma et al., 2016). Although all treatments were cultivated with the perennial plant (alfalfa) in our pots (Table 2), the components from fermented cow manure can affect rhizosphere microenvironments (Miethling et al., 2000), resulting in different bacterial community structures after the fertilization treatments. Overall, our results indicate that fertilization, especially residue of fermented cow manure (R_Manure) with a high C/N, has a significant impact on rhizosphere chemical properties and drive distinctly in bacterial community structures and co-occurrence networks.

### Distribution of bacterial taxa specific to the fertilization treatments

The sensitive OTUs (csOTUs) can be performed by a correlation-based indicator species analysis (Cáceres and Legendre, 2009). The 1,094 OTUs identified have strong and significant correlations with the fertilization treatments, while specific OTUs are prevalently distributed in their preferred treatments of fertilization. This information further suggested ecological attributes of these bacterial taxa that are sensitive to microenvironmental conditions of certain fertilization treatments. In particular, E_Manure had a much greater number of indicator OTUs (276) than other fertilization treatments except for the control with the indicator OTUs (316). Most of the indicator OTUs were associated with the phyla Actinobacteria, Bacteroidetes, Chloroflexi, Firmicutes, Proteobacteria, and so on. Phylum proteobacteria are the most widely distributed in all treatments and the phylum Firmicutes is the second most widely distributed in F_Manure, E_Manure, and R_Manure treatments. At the same time, the phylum Bacteroidetes is the third largest distribution with a relatively higher abundance except for the control treatments (Figure 4). Our results strongly support the copiotrophic hypothesis proposed by Fierer et al. (2007), which holds that bacteria with rapid growth rates (copiotrophs) tend to environments with high organic C and nutrient contents, while slow-growing bacteria (oligotrophs) may thrive in low-nutrient conditions. Therefore, nitrogen or organic fertilizers increased the abundance of the copiotrophic groups (e.g., Actinobacteria, Bacteroidetes, and Gammaproteobacteria) (Goldfarb et al., 2011). In this study, Bacteroidetes were positively correlated with the C/N contents but not to C or N content, on the contrary, Proteobacteria were negatively correlated with the C/N contents but not to C or N content. However, there is still a lot of work to be done to understand the ecological preference of those phyla.

The Chloroflexi detected in anaerobic environments are prevalent in oligotrophic environments (Janssen, 2006; Krzmarzick et al., 2012; Speirs et al., 2019), for example, in cow dung fermentation. For the cow dung, the fermentation provided anaerobic conditions and the residue of fermented cow manure (R_Manure) have higher OM and AN, while the abundances of Chloroflexi were relatively high in R_Manure, which were in keeping with previous studies (Fierer et al., 2012; Lee et al., 2015; Ahn et al., 2016; Hernández et al., 2017). Firmicutes were one of the abundant phyla not only in R_Manure but also in F_Manure indicator OTUs. Proteobacteria was another of the abundant phyla in all treatments and its abundant phyla are the least in indicator OTUs of R_Manure. Relationships between Firmicutes abundance and matrix chemical properties, such as carbon amendment level and pH, have been reported (Jones et al., 2009). This result is in keeping with our finding where Firmicutes had different distributions in the matrix of R_Manure and F_Manure. Many indicator OTUs in the matrix of R_Manure and F_Manure were associated with Firmicutes at a relatively higher abundance than that of the other treatments (Figure 5). Their high abundance in R_Manure and F_Manure, which contain relatively high levels of edaphic factors, is in keeping with previous studies (Leff et al., 2015; Trivedi et al., 2016). Especially in indicator OTUs of R_ manure, OTU404 (0.98%) related to Bacillus is the aggregation of Bacillus strains isolated from rhizosphere or endogenous sources such as alfalfa (Supplementary Table S2). Bacillus species is beneficial bacteria well known to promote plant growth and enhance plant tolerance to abiotic and biotic stresses (Baldani et al., 2014; Pujalte et al., 2014). Since alfalfa grows continuously in the matrix treated with R_Manure, lots of Bacilli closely related to this forage seem to be predominant in the matrix of R_Manure.

The 12 csOTUs identified were strongly related to sensitivity to C/N (Supplementary Table S2). They were assigned to Chloroflexi (OTU14, OTU4, OTU102, and OTU1104), Proteobacteria (OTU81, OTU1720, OTU629, OTU8420, and OTU143), Actinobacteria (OTU126), and Acidobacteria (OTU178). Caldilineaceae and A4b are families in the subclass Chloroflexi and comprise purple non-sulfur bacteria that are phototrophic in anaerobic environments (Baldani et al., 2014; Pujalte et al., 2014). Although C/N was one of the edaphic factors higher in the R_Manure and F_Manure, only one OTUs of C/N-sensitive OTUs were R_Manure-related indicator OTUs, indicating that in the condition of grassland growing alfalfa, complex factors in different fertilization treatments influence specific OTUs. Our results highlight the potential of these OTUs as applicable biological indicators for monitoring how matrix conditions are affected by the fertilization treatments. Although C/N was higher in R_Manure and F_Manure than in others, only one sensitive OTU related to R_Manure, which indicates that fertilization treatments influence specific OTUs. Our results highlight the potential of these csOTUs (e.g., phyla Chloroflexi, Acidobacteria, and Proteobacteria; order SBR1031, Caldilineales, Subgroup_6, Xanthomonadales, EPR3968-O8a-Bc78, Actinomarinales, and Alphaproteobacteria; and genus *MO-CFX2, SBR1031, Caldilineaceae, Subgroup_6, Lysobacter, EPR3968-O8a-Bc78, A4b, Actinomarinales, Alphaproteobacteria*, and *Alphaproteobacteria*) as biological indicators for monitoring manure fertilization treatments.

### Effects of fertilization treatments on co-occurrence networks

Co-occurrence networks were explored in four different treatments with components from fermented cow manure. Compared with the control, the microbial network of R_Manure with higher C/N has higher complexity. For example, R_Manure microbial network has more edges (105,692), more average degree (157.75), and low average clustering coefficient, which were two times higher than those of the control microbial network. Strong co-occurrence networks were also observed within the dominant phyla Acidobacteria, Bacteroidetes, and Proteobacteria.

The microbial interactions in networks show the structure and dynamics of rhizosphere microbial communities (Morlon et al., 2008; Tripathi et al., 2014; Hartmann et al., 2015). The indexes related to the co-occurrence networks and their topologies are clearly shown in the treatments of fertilization (Figure 6, Supplementary Table S3). Consistent with higher diversity in R_Manure, a relatively large number of OTUs were related to the networks attribute of R_Manure. Moreover, the number of links (ecount) was the highest in the topology attribute of R_Manure, reflecting multiple niches caused by the unique environmental feature of R_Manure. In contrast, the number of links (ecount) in the topology attribute of the Control was smaller so that the interactions of the networks were not close to each other (average degree is the lowest, Supplementary Table S3). As keystone taxa, Chloroflexi is the phylum enriched in R_Manure and F_Manure, which can be explained by similar chemical properties. The keystone taxa in R_Manure and F_Manure were associated with Gammaproteobacteria and Alphaproteobacteria, respectively, which were also the correspondingly rich phyla in these treatments compared with others. This result is consistent with the previous study that most OTUs related to microbial networks had a few links, although keystone taxa play an important role with a relatively lower abundance in microbial communities (Hartman et al., 2018). Our findings highlight the impact of organic fertilization on rhizosphere microbial health and sustainable alfalfa production.

## Conclusion

In summary, we showed that fertilization with different components from fermented cow manure affected matrix chemical properties, bacterial community structures, and co-occurrence networks, while distinctly enriched in specific taxa related to R_Manure and F_Manure. Furthermore, microbial interactions based on the co-occurrence patterns in rhizosphere bacterial communities also varied with different fertilization quality (C/N). Our findings provide a novel perspective of how to apply cow manure to improve the fertility for maintaining soil health and better alfalfa growth. These significant changes can be used as potential biological indicators to monitor the impact of grassland improvement management on the rhizosphere environment, while the composition of the rhizosphere microbial community is an important index to evaluate the environmental response of vegetation restoration.

## Data availability statement

The original contributions presented in the study are included in the article/Supplementary material, further inquiries can be directed to the corresponding author.

## Ethics statement

All procedures performed in the studies involving plant material were in accordance with the ethical standards of the institutional and/or national research committee. No specific permissions were required for the described field studies and for these locations/activities. The location is not privately owned or protected in any way. The studies did not involve endangered or protected species.

## Author contributions

RZ and SS designed the study. CL, JL, and RZ performed the testing. WH and YX performed the data analysis. JC and YA wrote the manuscript. RZ reviewed and approved the manuscript.

## Funding

This project was financially supported by the Natural Science Foundation of Heilongjiang Province (yq2019c019). Research on the construction of scientific and technological innovation system of germplasm resources (2021-DFZD-21-4), Chinese Academy of Engineering. The Special Project Personnel start-up Funds Academy of animal husbandry (22525), Chongqing Institute of Scientific Research.

## Conflict of interest

Author YA was employed by Gansu Yasheng Agricultural Research Institute Co., Ltd.

The remaining authors declare that the research was conducted in the absence of any commercial or financial relationships that could be construed as a potential conflict of interest.

## Publisher's note

All claims expressed in this article are solely those of the authors and do not necessarily represent those of their affiliated organizations, or those of the publisher, the editors and the reviewers. Any product that may be evaluated in this article, or claim that may be made by its manufacturer, is not guaranteed or endorsed by the publisher.

## References

[B1] Ahn J. H. Lee S. Kim J. M. Kim M. S. Song J. Weon H. Y. (2016). Dynamics of bacterial communities in rice field soils as affected by different long-term fertilization practices. J. Microbiol. 54, 724–731. 10.1007/s12275-016-6463-3 27796926

[B2] Aziz I. Mahmood T. Islam K. R. (2013). Effect of long term no-till and conventional tillage practices on soil quality. Soil Tillage Res. 131, 28–35. 10.1016/j.still.2013.03.00222762975

[B3] Baldani J. I. Videira S. S Teixeire K. R. S. Reis V. M. Oliveira A. L. M. Schwab S. (2014). “The Family Rhodospirillaceae,” in The Prokaryotes, ed E. Rosenberg (Berlin: Springer), 533–618. 10.1007/978-3-642-30197-1_300

[B4] Bao S. D. (2000). Soil and Agricultural Chemical Analysis. Beijing: China Agriculture Press. 10.4236/tel.2018.810124

[B5] Bartram A. K. Jiang X. Lynch M. D. Masella A. P. Nicol G. W. Dushoff J. (2014). Exploring links between pH and bacterial community composition in soils from the craibstone experimental farm. FEMS Microbiol. Ecol. 87, 403–415. 10.1111/1574-6941.12231 24117982

[B6] Bastian M. Heymann S. Jacomy M. (2009). “Gephi: an open source software for exploring and manipulating networks,” in Proceedings of the Third International AAAI Conference on Webblogs and Social Media (San Jose, CA), 361–362. 10.13140/2.1.1341.1520

[B7] Cáceres M. D. Legendre P. (2009). Associations between species and groups of sites: indices and statistical inference. Ecology 90, 3566–3574. 10.1890/08-1823.1 20120823

[B8] Chaudhry V. Rehman A. Mishra A. Chauhan P. S. Nautiyal C. S. (2012). Changes in bacterial community structure of agricultural land due to long-term organic and chemical amendments. Microb. Ecol. 64, 450–460. 10.2307/41692860 22419103

[B9] Chen J. S. Zhu R. F. Zhang Y. X. (2013). The effect of nitrogen addition on seed yield and yield components of *Leymus chinensis* in Songnen Plain, China. J. Soil Sci. Plant Nutr. 13, 329–339. 10.4067/S0718-95162013005000027

[B10] Davidson E. A. Janssens I. A. Luo Y. (2006). On the variability of respiration in terrestrial ecosystems: moving beyond Q10. Glob. Chang. Biol. 12, 154–164. 10.1111/j.1365-2486.2005.01065.x

[B11] Deng Y. Jiang Y. H. Yang Y. He Z. Luo F. Zhou J. (2012). Molecular ecological network analyses. BMC Bioinform. 13, 1–20. 10.1186/1471-2105-13-113 22646978 PMC3428680

[B12] Drenovsky R. E. Steenwerth K. L. Jackson L. E. Scow K. M. (2010). Land use and climatic factors structure regional patterns in soil microbial communities. Glob Ecol Biogeogr. 19, 27–39. 10.1111/j.1466-8238.2009.00486.x 24443643 PMC3891896

[B13] Fierer N. Bradford M. A. Jackson R. B. (2007). Toward an ecological classification of soil bacteria. Ecology 88, 1354–1364. 10.2307/27651243 17601128

[B14] Fierer N. Lauber C. L. Ramirez K. S. Zaneveld J. Bradford M. A. Knight R. (2012). Comparative metagenomic, phylogenetic and physiological analyses of soil microbial communities across nitrogen gradients. ISME J. 6, 1007–1017. 10.1038/ismej.2011.159 22134642 PMC3329107

[B15] Figuerola E. L. Guerrero L. D. Rosa S. M. Simonetti L. Duval M. E. Galantini J. A.. (2012). Bacterial indicator of agricultural management for soil under no-till crop production. PLoS ONE 7:e51075. 10.1371/journal.pone.0051075 23226466 PMC3511350

[B16] Goldfarb K. C. Karaoz U. Hanson C. A. Santee C. A. Bradford M. A. Treseder K. K.. (2011). Differential growth responses of soil bacterial taxa to carbon substrates of varying chemical recalcitrance. Front Microbiol. 2:94. 10.3389/fmicb.2011.00094 21833332 PMC3153052

[B17] Handley K. M. Wrighton K. C. Piceno Y. M. Andersen G. L. DeSantis T. Z. Williams K. H. (2012). High-density PhyloChip profiling of stimulated aquifer microbial communities reveals a complex response to acetate amendment. FEMS Microbiol. Ecol. 81, 188–204. 10.1111/j.1574-6941.2012.01363.x 22432531

[B18] Hartman K. van der Heijden M. G. Wittwer R. A. Banerjee S. Walser J. C. Schlaeppi K. (2018). Cropping practices manipulate abundance patterns of root and soil microbiome members paving the way to smart farming. Microbiome 6, 1–14. 10.1186/s40168-017-0389-9 29338764 PMC5771023

[B19] Hartmann M. Frey B. Mayer J. Mäder P. Widmer F. (2015). Distinct soil microbial diversity under long-term organic and conventional farming. ISME J. 9, 1177–1194. 10.1038/ismej.2014.210 25350160 PMC4409162

[B20] Heijden M. Bardgett R. D. Straalen N. (2008). The unseen majority: soil microbes as drivers of plant diversity and productivity in terrestrial ecosystems. Ecol. Lett. 11, 296–310. 10.1111/j.1461-0248.2007.01139.x 18047587

[B21] Hernández M. Conrad R. Klose M. Ma K. Lu Y. (2017). Structure and function of methanogenic microbial communities in soils from flooded rice and upland soybean fields from Sanjiang plain, NE China. Soil Biol. Biochem. 105, 81–91. 10.1016/j.soilbio.2016.11.010

[B22] Janssen P. H. (2006). Identifying the dominant soil bacterial taxa in libraries of 16S rRNA and 16S rRNA genes. Appl. Environ. Microbiol. 72, 1719–1728. 10.1128/aem.72.3.1719-1728.2006 16517615 PMC1393246

[B23] Jiménez-Bueno N. G. Valenzuela-Encinas C. Marsch R. Ortiz-Gutiérrez D. Verhulst N. Govaerts B.. (2016). Bacterial indicator taxa in soils under different long-term agricultural management. J. Appl. Microbiol. 120, 921–933. 10.1111/jam.13072 26808352

[B24] Jones R. T. Robeson M. S. Lauber C. L. Hamady M. Knight R. Fierer N. (2009). A comprehensive survey of soil acidobacterial diversity using pyrosequencing and clone library analyses. ISME J. 3, 442–453. 10.1038/ismej.2008.127 19129864 PMC2997719

[B25] Kang S. S. Roh A. S. Choi S. C. Kim Y. S. Kim H. J. Choi M. T.. (2013). Status and change in chemical properties of polytunnel soil in Korea from 2000 to 2012. Kor. J. Soil Sci. Fertil. 46, 641–646. 10.7745/KJSSF.2013.46.6.641S

[B26] Kennedy A. C. (1999). Bacterial diversity in agroecosystems. Agric. Ecosyst. Environ. 74, 65–76. 10.1016/S0167-8809(99)00030-4

[B27] Krzmarzick M. J. Crary B. B. Harding J. J. Oyerinde O. O. Leri A. C. Myneni S. C.. (2012). Natural niche for organohalide-respiring Chloroflexi. Appl. Environ. Microbiol. 78, 393–401. 10.1128/AEM.06510-11 22101035 PMC3255752

[B28] Lauber C. L. Strickland M. S. Bradford M. A. Fierer N. (2008). The influence of soil properties on the structure of bacterial and fungal communities across land-use types. Soil Biol. Biochem. 40, 2407–2415. 10.1016/j.soilbio.2008.05.021

[B29] Lee H. J. Jeong S. E. Kim P. J. Madsen E. L. Jeon C. O. (2015). High resolution depth distribution of Bacteria, Archaea, methanotrophs, and methanogens in the bulk and rhizosphere soils of a flooded rice paddy. Front. Microbiol. 6:639. 10.3389/fmicb.2015.00639 26161079 PMC4479796

[B30] Leff J. W. Jones S. E. Prober S. M. Barberán A. Borer E. T. Firn J. L.. (2015). Consistent responses of soil microbial communities to elevated nutrient inputs in grasslands across the globe. Proc. Natl. Acad. Sci. U.S.A. 112, 10967–10972. 10.1073/pnas.1508382112 26283343 PMC4568213

[B31] Liesack W. Schnell S. Revsbech N. P. (2000). Microbiology of flooded rice paddies. FEMS Microbiol. Rev. 24, 625–645. 10.1111/j.1574-6976.2000.tb00563.x 11077155

[B32] Ma B. Dai Z. Wang H. Dsouza M. Liu X. He Y.. (2017). Distinct biogeographic patterns for archaea, bacteria, and fungi along the vegetation gradient at the continental scale in Eastern China. Msystems 2:e00174-16. 10.1128/msystems.00174-16 28191504 PMC5296412

[B33] Ma J. Ibekwe A. M. Yang C. H. Crowley D. E. (2016). Bacterial diversity and composition in major fresh produce growing soils affected by physiochemical properties and geographic locations. Sci. Total Environ. 563, 199–209. 10.1016/j.scitotenv.2016.04.122 27135583

[B34] Marschner P. Crowley D. Yang C. H. (2004). Development of specific rhizosphere bacterial communities in relation to plant species, nutrition and soil type. Plant Soil. 261, 199–208. 10.1023/B:PLSO.0000035569.80747.c5

[B35] Mendes R. Garbeva P. Raaijmakers J. M. (2013). The rhizosphere microbiome: significance of plant beneficial, plant pathogenic, and human pathogenic microorganisms. FEMS Microbiol. Rev. 37, 634–663. 10.1111/1574-6976.12028 23790204

[B36] Meriles J. M. Gil S. V. Conforto C. Figoni G. Lovera E. March G. J.. (2009). Soil microbial communities under different soybean cropping systems: characterization of microbial population dynamics, soil microbial activity, microbial biomass, and fatty acid profiles. Soil Tillage Res. 103, 271–281. 10.1016/j.still.2008.10.008

[B37] Miethling R. Wieland G. Backhaus H. Tebbe C. C. (2000). Variation of microbial rhizosphere communities in response to crop species, soil origin, and inoculation with *Sinorhizobium meliloti* L33. Microb. Ecol. 40, 43–56. 10.1007/s002480000021 10977876

[B38] Min W. Guo H. Zhang W. Zhou G. Ma L. Ye J. (2016). Response of soil microbial community and diversity to increasing water salinity and nitrogen fertilization rate in an arid soil. Acta Agric. Scand. B Soil Plant Sci. 66, 117–126. 10.1080/09064710.2015.1078838

[B39] Morlon H. Chuyong G. Condit R. Hubbell S. Kenfack D. Thomas D.. (2008). A general framework for the distance–decay of similarity in ecological communities. Ecol. Lett. 11, 904–917. 10.1111/j.1461-0248.2008.01202.x 18494792 PMC2613237

[B40] Navarro-Noya Y. E. Gómez-Acata S. Montoya-Ciriaco N. Rojas-Valdez A. Suárez-Arriaga M. C. Valenzuela-Encinas C.. (2013). Relative impacts of tillage, residue management and crop-rotation on soil bacterial communities in a semi-arid agroecosystem. Soil Biol. Biochem. 65, 86–95. 10.1016/j.soilbio.2013.05.009

[B41] Ogle D. H. (2017). FSA: Fisheries Stock Analysis. R Package Version 0.8.13.

[B42] Osler G. H. Sommerkorn M. (2007). Toward a complete soil C and N cycle: incorporating the soil fauna. Ecology 88, 1611–1621. 10.1890/06-1357.1 17645007

[B43] Pujalte M. J. Lucena T. Ruvira M. A. Arahal D. R. Macián M. C. (2014). “The Family *Rhodobacteraceae*,” In, The Prokaryotes, eds E. Rosenberg, E. F. DeLong, S. Lory, E. Stackebrandt, and F. Thompson (Berlin; Heidelberg: Springer), 439–512. 10.1007/978-3-642-30197-1_377

[B44] R Core Team. (2013). R: A Language and Environment for Statistical Computing. 10.1890/0012-9658(2002)083

[B45] Reich P. B. Oleksyn J. Modrzynski J. Mrozinski P. Hobbie S. E. Eissenstat D. M.. (2005). Linking litter calcium, earthworms and soil properties: a common garden test with 14 tree species. Ecol. Lett. 8, 811–818. 10.1111/j.1461-0248.2005.00779.x

[B46] Sala O. E. Stuart Chapin F. III Armesto J. J. Berlow E. Bloomfield J. Dirzo R.. (2000). Global biodiversity scenarios for the year 2100. Science 287, 1770–1774. 10.2307/3074591 10710299

[B47] Shen C. C. Xiong J. B. Zhang H. Y. Feng Y. Z. Lin X. G. Li X. Y.. (2013). Soil ph drives the spatial distribution of bacterial communities along elevation on changbai mountain. Soil Biol. Biochem. 57, 204–211. 10.1016/j.soilbio.2012.07.013

[B48] Singh D. Shi L. Adams J. M. (2013). Bacterial diversity in the mountains of south-west China: climate dominates over soil parameters. J. Microbiol. 51, 439–447. 10.1007/s12275-013-2446-9 23990294

[B49] Singh J. S. Pandey V. C. Singh D. P. (2011). Efficient soil microorganisms: a new dimension for sustainable agriculture and environmental development. Agric. Ecosyst. Environ. 140, 339–353. 10.1016/j.agee.2011.01.017

[B50] Song X. P. Hansen M. C. Stehman S. V. Potapov P. V. Tyukavina A. Vermote E. F.. (2018). Global land change from 1982 to 2016. Nature 560, 639–643. 10.1038/s41586-018-0411-9 30089903 PMC6366331

[B51] Speirs L. B. Rice D. T. Petrovski S. Seviour R. J. (2019). The phylogeny, biodiversity, and ecology of the Chloroflexi in activated sludge. Front. Microbiol. 10:2015. 10.3389/fmicb.2019.02015 31572309 PMC6753630

[B52] Sridevi G. Minocha R. Turlapati S. A. Goldfarb K. C. Brodie E. L. Tisa L. S.. (2012). Soil bacterial communities of a calcium-supplemented and a reference watershed at the Hubbard Brook Experimental Forest (HBEF), New Hampshire, USA. FEMS Microbiol. Ecol. 79, 728–740. 10.1111/j.1574-6941.2011.01258.x 22098093

[B53] Tripathi B. M. Lee-Cruz L. Kim M. Singh D. Go R. Shukor N. A.. (2014). Spatial scaling effects on soil bacterial communities in Malaysian tropical forests. Microb. Ecol. 68, 247–258. 10.1007/s00248-014-0404-7 24658414

[B54] Tripathi B. M. Stegen J. C. Kim M. Dong K. Adams J. M. Lee Y. K. (2018). Soil pH mediates the balance between stochastic and deterministic assembly of bacteria. ISME J. 12, 1072–1083. 10.1038/s41396-018-0082-4 29515169 PMC5864241

[B55] Trivedi P. Delgado-Baquerizo M. Anderson I. C. Singh B. K. (2016). Response of soil properties and microbial communities to agriculture: implications for primary productivity and soil health indicators. Front. Plant Sci. 7:990. 10.3389/fpls.2016.00990 27462326 PMC4940416

[B56] Verhulst N. Kienle F. Sayre K. D. Deckers J. Raes D. Limon-Ortega A.. (2011). Soil quality as affected by tillage-residue management in a wheat-maize irrigated bed planting system. Plant Soil. 340, 453–466. 10.1007/s11104-010-0618-5

[B57] Walther G. R. Post E. Convey P. Menzel A. Parmesan C. Beebee T. J.. (2002). Ecological responses to recent climate change. Nature 416, 389–395. 10.1038/416389a 11919621

[B58] Wang X. B. Lü X. T. Yao J. Wang Z. W. Deng Y. Cheng W. X. (2017). Habitat-specific patterns and drivers of bacterial β-diversity in China's drylands. ISME J. 11, 1345–1358. 10.1038/ismej.2017.11 28282041 PMC5437346

[B59] Wang Y. Ji H. Gao C. (2016). Differential responses of soil bacterial taxa to long-term P, N, and organic manure application. J.Soils Sediments 16, 1046–1058. 10.1007/s11368-015-1320-2

[B60] Yang Y. Dou Y. An S. (2018). Testing association between soil bacterial diversity and soil carbon storage on the Loess Plateau. Sci. Total Environ. 626, 48–58. 10.1016/j.scitotenv.2018.01.081 29335174

[B61] Yang Y. Gao Y. Wang S. Xu D. Yu H. Wu L. (2014). The microbial gene diversity along an elevation gradient of the Tibetan grassland. ISME J. 8, 430–440. 10.1038/ismej.2013.146 23985745 PMC3906809

[B62] Zhang Q. Shan L. Kong X. Li D. Kang X. Zhu R.. (2018). Effects of clipping frequency on the relationships between species diversity and productivity in temperate steppe. Int. J. Agric. Biol. 20, 2325–2328. 10.17957/IJAB/15.0803

[B63] Zhang Y. Cong J. Lu H. Deng Y. Liu X. Zhou J.. (2016). Soil bacterial endemism and potential functional redundancy in natural broadleaf forest along a latitudinal gradient. Sci. Rep. 6, 1–8. 10.1038/srep28819 27357005 PMC4928066

[B64] Zhang Y. Cong J. Lu H. Li G. Xue Y. Deng Y.. (2015). Soil bacterial diversity patterns and drivers along an elevational gradient on Shennongjia Mountain, China. Microb. Biotechnol. 8, 739–746. 10.1111/1751-7915.12288 26032124 PMC4476828

[B65] Zhang Y. Liu X. Cong J. Lu H. Sheng Y. Wang X.. (2017). The microbially mediated soil organic carbon loss under degenerative succession in an alpine meadow. Mol. Ecol. 26, 3676–3686. 10.1111/mec.14148 28429833

[B66] Zhou J. Bruns M. A. Tiedje J. M. (1996). DNA recovery from soils of diverse composition. Appl. Environ. Microbiol. 62, 316–322. 10.1128/aem.62.2.316-322.1996 8593035 PMC167800

[B67] Zhou J. Deng Y. Luo F. He Z. Tu Q. Zhi X. (2010). Functional molecular ecological networks. MBio 1:e00169-10. 10.1128/mBio.00169-10 20941329 PMC2953006

[B68] Zhou J. Deng Y. Luo F. He Z. Yang Y. (2011). Phylogenetic molecular ecological network of soil microbial communities in response to elevated CO_2_. MBio 2:e00122-11. 10.1128/mBio.00122-11 21791581 PMC3143843

[B69] Zhu R. Liu J. Wang J. Han W. Shen Z. Muraina T. O.. (2020). Comparison of soil microbial community between reseeding grassland and natural grassland in Songnen Meadow. Sci. Rep. 10, 1–11. 10.1038/s41598-020-74023-x 33037306 PMC7547709

