# Supplementary material for: Ratio of carbon and nitrogen in fertilizer treatment drives distinct rhizosphere microbial community composition and co-occurrence networks
Source: Front Microbiol. 2022 Sep 8;13:968551. doi: 10.3389/fmicb.2022.968551 (PMC9493311; doi:10.3389/fmicb.2022.968551)
Supplement: Supplementary file 1 [file Data_Sheet_1.docx]

**Supplementary Information**

**Different ratio of carbon and nitrogen drives distinct soil bacterial community composition and co‑occurrence networks**

**Authors:** Ruifen Zhu^1,2,3^, Chang Liu^1^, Yuan dong Xu^2^, Wei He^2^, Jielin Liu^3^, Jishan Chen^2^, Yajun An^4^, Shangli Shi^1*^

**Affiliations:**

^1^Pratacultural College, Gansu Agricultural University, Lanzhou, 730070, China;

^2^Pratacultural Institute, Chongqing Academy of Animal Sciences, Rongchang, 402460, China;

^3^Pratacultural Institute Science, Heilongjiang Academy of Agricultural Sciences, Harbin, 150086, China;

^4^Gansu Yasheng Agricultural Research Institute Co., Ltd, Lanzhou, 730070, China;

**^*^Correspondign Author:** Shang-Li Shi (shishl@gsau.edu.cn)


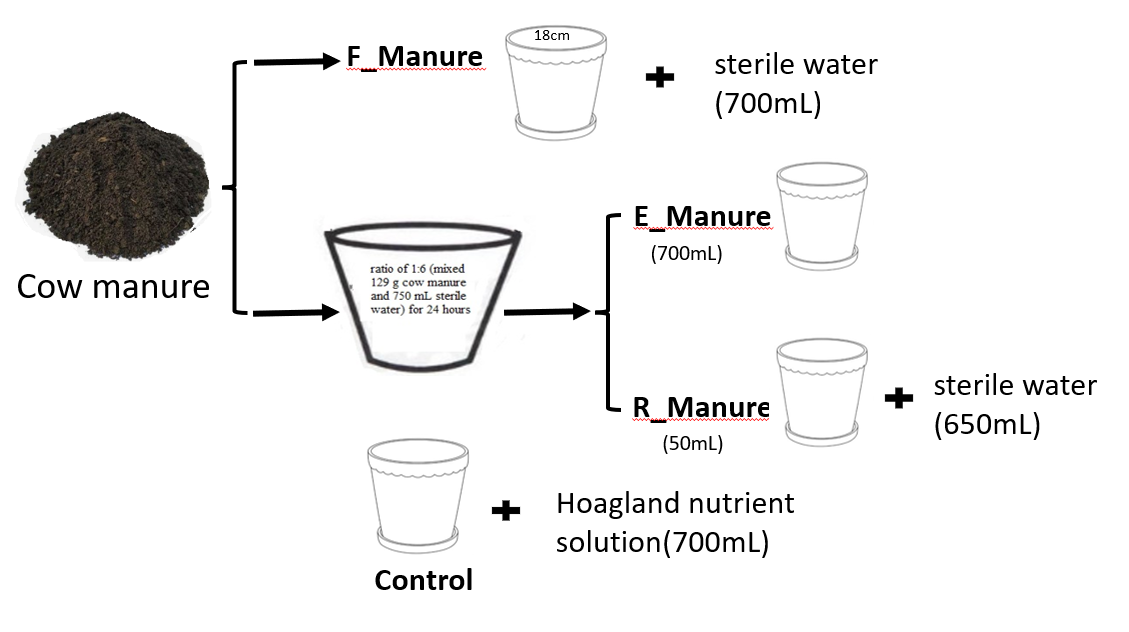
**Supplementary Figure S1.** Experimental design and treatment description including the randomized block trials with four treatments (Bold black type) and 12 replicates for each treatment. Hoagland nutrient solution was made up of those (6 mM KNO_3_; 4 mM Ca(NO_3_)_2_·4 H_2_O; 2 mM MgSO_4_·7 H_2_O; 1 mM (NH_4_)_2_PO_4_; 50 μM KCl, 25 μM H_3_BO_3_; 2 μM MnSO_4_·H_2_O; 2 μM ZnSO_4_·7 H_2_O; 0.3 μM CuSO_4_·5 H_2_O; 0.5 μM H_2_MoO_4_, 20 μM Fe-EDTA). The control was to be comprised of the equal portions (3.5 kg with a volume of 0.0028 m^3^) of sands measured and the Hoagland nutrient solution sprayed with a certain amount of 700 ml per plastic flowerpot (a volume of 0.0028 m^3^).

**Supplementary Table S1.** Pair-wise comparison between the treatments of fertilization based on bacterial communities.

|  | ANOSIM | | MRPP | | PERMANOVA | |
| --- | --- | --- | --- | --- | --- | --- |
| Group | R | P | A | P | R^2^ | P |
| Cont/F_Manure | 0.6806 | 0.0031 | 0.0382 | 0.006 | 0.0115 | 0.001 |
| Cont/E_Manure | 0.4167 | 0.0018 | 0.0311 | 0.001 | 0.0280 | 0.004 |
| Cont/R_Manure | 0.3305 | 0.0036 | 0.0405 | 0.014 | 0.0442 | 0.008 |
| F_Manure/E_Manure | 0.3821 | 0.0018 | 0.0323 | 0.043 | 0.0143 | 0.001 |
| F_Manure/R_Manure | 0.1029 | 0.0039 | 0.0621 | 0.003 | 0.0243 | 0.008 |
| E_Manure/R_Manure | 0.3104 | 0.0019 | 0.0299 | 0.038 | 0.0434 | 0.009 |

ANOSIM, analysis of similarities; MRPP, multiple response permutation procedure; PERMANOVA, permutational multivariate analysis of variance.

**Supplementary Table S2.** csOTU of the networks of bacterial communities in four different treatments. The csOTU with highest degree indicate potential keystone taxa in the complex microbial interactions.

| csOTU | Kingdom | Phylum | Class | Order | Family | Genus | Degree |
| --- | --- | --- | --- | --- | --- | --- | --- |
| OTU178 | Bacteria | Acidobacteria | Subgroup_6 | uncultured_bacterium_c_Subgroup_6 | uncultured_bacterium_c_Subgroup_6 | uncultured_bacterium_c_Subgroup_6 | 174 |
| OTU126 | Bacteria | Actinobacteria | Acidimicrobiia | Actinomarinales | uncultured_bacterium_o_Actinomarinales | uncultured_bacterium_o_Actinomarinales | 170 |
| OTU14 | Bacteria | Chloroflexi | Anaerolineae | SBR1031 | anaerobic_bacterium_MO-CFX2 | uncultured_bacterium_f_anaerobic_bacterium_MO-CFX2 | 130 |
| OTU4 | Bacteria | Chloroflexi | Anaerolineae | SBR1031 | uncultured_bacterium_o_SBR1031 | uncultured_bacterium_o_SBR1031 | 127 |
| OTU1104 | Bacteria | Chloroflexi | Anaerolineae | Caldilineales | Caldilineaceae | uncultured_bacterium_f_Caldilineaceae | 174 |
| OTU102 | Bacteria | Chloroflexi | Anaerolineae | SBR1031 | A4b | uncultured_bacterium_f_A4b | 170 |
| OTU404 | Bacteria | Firmicutes | Bacilli | Bacillales | Paenibacillaceae | Aneurinibacillus | 176 |
| OTU1700 | Bacteria | Proteobacteria | Deltaproteobacteria | NB1-j | uncultured_bacterium_o_NB1-j | uncultured_bacterium_o_NB1-j | 176 |
| OTU81 | Bacteria | Proteobacteria | Gammaproteobacteria | Xanthomonadales | Xanthomonadaceae | Lysobacter | 172 |
| OTU629 | Bacteria | Proteobacteria | Gammaproteobacteria | EPR3968-O8a-Bc78 | uncultured_bacterium_o_EPR3968-O8a-Bc78 | uncultured_bacterium_o_EPR3968-O8a-Bc78 | 171 |
| OTU8420 | Bacteria | Proteobacteria | Alphaproteobacteria | uncultured_bacterium_c_Alphaproteobacteria | uncultured_bacterium_c_Alphaproteobacteria | uncultured_bacterium_c_Alphaproteobacteria | 170 |
| OTU143 | Bacteria | Proteobacteria | Alphaproteobacteria | uncultured_bacterium_c_Alphaproteobacteria | uncultured_bacterium_c_Alphaproteobacteria | uncultured_bacterium_c_Alphaproteobacteria | 168 |

**Supplementary Table S3.** Topological properties of the networks of bacterial communities in four different fertilization treatments.

| Network indices | Cont | F_Manure | E_Manure | R_Manure |
| --- | --- | --- | --- | --- |
| No. of nodes (vcount) | 1111 | 1254 | 1396 | 1340 |
| No. of links (ecount) | 40479 | 58967 | 87693 | 105692 |
| Average path length | 3.83 | 3.60 | 3.55 | 3.04 |
| Graph density | 0.07 | 0.08 | 0.09 | 0.12 |
| Diameter | 17.00 | 14.00 | 14.00 | 12.00 |
| Average degree (avgK) | 72.87 | 94.05 | 125.63 | 157.75 |

**Supplementary Table S4.** Keystone taxa in the co-occurrence networks of bacterial communities in four different treatments. The OTUs with highest degree indicate potential keystone taxa in the complex microbial interactions.

|  | Kingdom | Phylum | Class | Order | Family | Genus | Degree |
| --- | --- | --- | --- | --- | --- | --- | --- |
| OTU14 | Bacteria | Chloroflexi | Anaerolineae | SBR1031 | anaerobic_bacterium_MO-CFX2 | uncultured_bacterium_f_anaerobic_bacterium_MO-CFX2 | 130 |
| OTU4 | Bacteria | Chloroflexi | Anaerolineae | SBR1031 | uncultured_bacterium_o_SBR1031 | uncultured_bacterium_o_SBR1031 | 127 |
| OTU1104 | Bacteria | Chloroflexi | Anaerolineae | Caldilineales | Caldilineaceae | uncultured_bacterium_f_Caldilineaceae | 174 |
| OTU178 | Bacteria | Acidobacteria | Subgroup_6 | uncultured_bacterium_c_Subgroup_6 | uncultured_bacterium_c_Subgroup_6 | uncultured_bacterium_c_Subgroup_6 | 174 |
| OTU81 | Bacteria | Proteobacteria | Gammaproteobacteria | Xanthomonadales | Xanthomonadaceae | Lysobacter | 172 |
| OTU629 | Bacteria | Proteobacteria | Gammaproteobacteria | EPR3968-O8a-Bc78 | uncultured_bacterium_o_EPR3968-O8a-Bc78 | uncultured_bacterium_o_EPR3968-O8a-Bc78 | 171 |
| OTU102 | Bacteria | Chloroflexi | Anaerolineae | SBR1031 | A4b | uncultured_bacterium_f_A4b | 170 |
| OTU126 | Bacteria | Actinobacteria | Acidimicrobiia | Actinomarinales | uncultured_bacterium_o_Actinomarinales | uncultured_bacterium_o_Actinomarinales | 170 |
| OTU8420 | Bacteria | Proteobacteria | Alphaproteobacteria | uncultured_bacterium_c_Alphaproteobacteria | uncultured_bacterium_c_Alphaproteobacteria | uncultured_bacterium_c_Alphaproteobacteria | 170 |
| OTU143 | Bacteria | Proteobacteria | Alphaproteobacteria | uncultured_bacterium_c_Alphaproteobacteria | uncultured_bacterium_c_Alphaproteobacteria | uncultured_bacterium_c_Alphaproteobacteria | 168 |
